# A Study of Family Process Factors of Social Anxiety on the Internet Based on Big Data—Take Guangxi University Students as an Example

**DOI:** 10.3389/fpubh.2022.870822

**Published:** 2022-03-29

**Authors:** Dexu Bi

**Affiliations:** Department of Elementary Education, Guangxi Police College, Nanning, China

**Keywords:** family systems theory, Guangxi University students, online social anxiety, family process factors, big data

## Abstract

**Background:**

Along with the popularization of the new medium of interpersonal communication, many researchers have found that the use of social media has brought about many mental health problems. For example, the virtual nature, vulnerability, and uncertainty of online communication lead to reduced online trust, causing interaction anxiety (IA). The data footprints left on the Internet are processed by malicious elements for big data, leading to the leakage of personal privacy data, bringing content sharing anxiety (SAC) and privacy concern anxiety (PAC), which are all typical forms of online social anxiety. In the face of this situation, analyzing the influence of online social networking on the social psychology of university students and guiding it has become an inevitable issue in the Internet era.

**Methods:**

Learning from the classification of family environment, a self-administered family process factor questionnaire and the Social Anxiety Scale for Social Media Users (SAS-SMU) were used to investigate the online social anxiety of Guangxi University students. The study used SPSS26.0 and Stata for data analysis and descriptive statistics, ANOVA, *t*-test, and linear regression analysis were used to explore the relationship between family process factors and online social anxiety of the university students.

**Results:**

The results showed that except for parental supervision (*p* > 0.05), the effects of interparental relationship, parent-child relationship, sibling relationship, and family atmosphere on university students' online social anxiety were statistically significant and showed positive correlations (F/t = 6.64, 3.53, 4.15, 5.94; *p* < 0.05). Multiple linear regression analysis showed that university students' total online social anxiety score = 36.914−4.09 × good parental relationship−4.16 × good family atmosphere−3.42 × good sibling relationship.

**Conclusions:**

Based on the family systems theory, it is suggested that a comprehensive intervention should be conducted for the coupled system (parental relationship) and sibling system (non-only child's sibling relationship) in the family and focus on the protective factors of parental harmony, sibling relationship harmony, and relaxed family atmosphere. In the specific implementation method, the collaborative shared healthcare plan (CSHCP) can be used to strengthen remote family emotional interaction and avoid Internet addiction. For university students with online social anxiety disorders, their personal health records (PHRs) can be maintained permanently and safely using the Star File System (IPFS), in addition to the convenience of IPFS data extraction, which is more conducive to the timely and long-term tracking treatment of anxious university students.

## Introduction

With the development of the Internet, there are at least two different forms of modern human interaction activities, namely, real face-to-face interaction in the traditional social presence space and non-face-to-face network interaction in the absence of field cyberspace. As a new form of social interaction, network interaction is changing the way people interact socially and has become an important way of interpersonal communication. The 2020 Digital Report, published by We Are Social, shows that more than 4.5 billion people use the Internet, and social media users have surpassed 3.8 billion (1). According to the 46th Statistical Report on the Development Status of the Internet in China released by the China Internet Network Information Center (CNNIC), Chinese Internet users reached 940 million by June 2020. The scale of instant messaging users reached 99.0% of the overall Internet users (2). It can be said that socializing through the Internet has become the most important form of human interaction.

Along with the popularization of the new medium of interpersonal communication, many researchers have found that the use of social media has brought about many mental health problems. For example, the virtual nature, vulnerability, and uncertainty of online communication lead to reduced online trust, causing interaction anxiety (IA). Social networking data is maliciously collected and leaked by people with the help of big data technology, resulting in content sharing anxiety (SAC) and privacy concern anxiety (PAC) among network users. Online social anxiety is a significant and persistent emotional response of worry, fear, and avoidance behavior to one or more interpersonal situations in the interpersonal exchange of information and emotions based on the Internet and mediated by computers (3). Basic performance behaviors are fear of interacting with others, fear of embarrassing oneself with the content shared, fear of chatting with others online, and fear of others making negative comments about oneself because of the content shared, etc.

As the main group of social media users, university students are troubled by “uncertainty,” “social addiction,” and privacy leakage while gaining access to various convenient services brought by the Internet, thus, giving rise to online social anxiety. In the face of this situation, it has become an inevitable problem to analyze the influencing factors of university students' online social networking and guide it. As a result, this article will select university students in Guangxi as the research object and take family process factors as the link to empirically analyze the family process factors of online social anxiety in university students in order to provide guidance for the treatment and behavioral improvement of online social anxiety.

### Progress of Research on Social Anxiety in Adolescents Guided by Family Systems Theory

American scholar M. Bowen and his collaborator M.E. Kerr's family systems theory (4, 5) considers the family as a “grouped emotional body” that is the result of the interaction between the “father-mother-child” triangle in which individuality or separateness and togetherness or fusion are intertwined—individuality (or separateness) and togetherness (or fusion). Individuality and togetherness are two important balancing agents in the family system. The former seeks independence, while the latter seeks family belonging. Ideally, the family relationship is one in which these two forces are in balance. In this case, family members have their own independence and maintain close ties with each other. Suppose the family relationship is too close and the children are too emotionally involved with their families. In that case, the children will have low levels of Emotional Fusion and Differentiation and will be less independent. Conversely, if they are too distant, they have high levels of Emotional Fusion and Differentiation and are too independent. Both of these imbalance models produce dysregulation of the nuclear family emotional management system, where children need to pay constant attention to their parents' demands and emotional performance, are unable to think and act according to their own needs, are under high tension for long periods of time, have excessive emotional stress intensity and become increasingly attached to becoming independent, resulting in interpersonal difficulties within the family. Suppose individuals respond to the emotional needs of others by choosing to cluster or choosing Emotional Fusion and Differentiation in a fragmented way. In that case, they will develop anxiety and show anxious behavior.

Bowen's family systems theory has provided new research ideas for adolescent clinical psychology research. Based on this theory, many scholars have examined the mechanisms by which family systems interact with each other in the psychogenesis and development of adolescents, especially providing a new analytical framework for exploring the influence of parent-child relationship quality on the psychosocial adjustment of family members. Drawing on Bowen's family systems theory, Williams (6) further generalized the family environment into non-process and process factors based on the presence or absence of family members' interactions. The non-process factors refer to the members' own characteristics or inherent characteristics of the family environment, which mainly include static factors, such as family structure, family socioeconomic status, primary provider, primary caregiver, and parents' own characteristics (7). Process factors refer to the interactions between members within the family system and the family climate formed by the interactions that mainly include the couple relationship, parenting, parent-child relationship, non-only child sibling relationship, and the family climate formed by them.

Chinese and foreign scholars have conducted a large number of studies on the effects of family non-process factors on social anxiety. They mainly involve the effects of gender (8, 9), family structure (10), grade level (11), and family economic and social status (12, 13) on social anxiety. The influence on family process factors involves parenting style (14, 15), family closeness (16), family function (17, 18), and parental conflict (19–22). Compared to the non-process factors that influence social anxiety, which are difficult to change, the process factors can be effectively guided and have more plastic value for the benign development of social anxiety. However, from the general status quo of research on family factors of social anxiety at this stage, family process factors are either done as single-factor research, which is in-depth but lacks systematicity, or put into family factors as grand unified research, which does not make specialized distinctions and lacks in-depth insight. Specifically, no directly relevant domestic and international literature has been retrieved on the empirical study of helping university students overcome online social anxiety through the improvement of family process factors.

## Materials and Methods

### Study Participants

From September 2021 to October 2021, Guangxi Police College, Guangxi Foreign Language Institute, Nanning College, and Guangxi International Business Vocational and Technical College were selected. The anonymous questionnaire test was conducted with the informed consent of the subjects, and the information of the subjects was kept strictly confidential. A total of 2,400 questionnaires were distributed, and 2,373 valid questionnaires were returned, with a valid recovery rate of 94.6%. The age range was 18–22 years old, with 1,125 men (47.4%) and 1,248 women (52.6%). There were 995 people from rural areas, accounting for 41.9%, and 1,378 people from urban areas, accounting for 58.1%. As for the structure of majors, 1,350 students were studying literature, history, and finance, and 1,023 students were studying science and agriculture, accounting for 56.9 and 43.1%, respectively. In terms of grade structure, the numbers of freshmen, sophomores, juniors, and seniors were 503, 657, 797, and 416, accounting for 21.2, 27.7, 33.6, and 17.5%.

### Research Method

The online social anxiety scale was adopted from the Social Anxiety Scale for Social Media Users (SAS-SMU) revised by Wang (23), which was validated and revised based on the online SAS-SMU established by Alkis et al. in Turkey. It consists of three dimensions with 15 entries, and the three dimensions are SAC, PAC, and IA. The 5-point Likert scale was used (not at all, rarely, sometimes, often, and completely), with higher scores indicating a more severe degree of online social anxiety (24). The Cronbach α = 0.928 in this survey.

Family process factors include (1) parent-child relationships (poor, fair, and good), and problem behavior theory suggests that a good parent-child relationship will increase their regular behaviors with a corresponding decrease in problem behaviors. (2) Interparental relationships (poor, fair, and good), family dynamics theory suggests that if an individual is in a deteriorating family relationship, it will lead to poor socialization of the child (25). (3) Family climate (poor, fair, and good), research has shown that the more harmonious the family climate is perceived by adolescents, the less they engage in problem behaviors. (4) Parental supervision (rarely, occasionally, and often), according to Jessor's problem behavior theory, the higher the level of support and control (especially from parents) perceived by adolescents, the less likely they are to engage in problem behaviors (26). (5) Sibling relationship (poor, fair, and good).

### Data Collection

The survey was conducted by the Public Foundation Department of Guangxi Police College and some students used the actual test. Before the survey, the investigators were trained in a unified manner. In the survey, arranged quality control personnel in the field are responsible for answering possible problems to the survey respondents in a timely manner. The surveyors are responsible for recovering and reviewing the completeness of the completed questionnaires (27). The study used SPSS 26.0 and Stata for data analysis; descriptive statistics, one-way ANOVA, *t*-test, and linear regression analysis were used to explore the relationship between family process factors and online social anxiety of university students. *P* < 0.05 indicates that the difference is statistically significant.

## Statistical Analysis

### Descriptive Analysis of Family Process Factors of Guangxi University Students

There were 322 (13.6%) only children and 2,051 (86.4%) non-only children among 2,373 university students. The numbers of non-only children's families with poor, fair, and good sibling relationships were 29 (1.41%), 317 (15.5%), and 1,720 (83.1%). The numbers of poor, moderate, and good parental relationships were 161 (6.78%), 640 (27%), and 1,572 (66.25%). The frequency of family supervision was rarely, occasionally, and often for 72 (3%), 1,520 (64.1%), and 781 (32.9%). Those with poor, average, and good parent-child relationships were 31 (1.3%), 692 (29.2%), and 1,650 (69.5%). Poor, fair, and good family atmospheres occurred in 531 (22.4%), 1,330 (56%), and 512 (21.6%).

### Differential Analysis of Family Process Factors of Online Social Anxiety

Table 1 shows, except for parental supervision (*p* > 0.05), that the effects of interparental relationship, parent-child relationship, sibling relationship, and family atmosphere on online social anxiety in rural university students were statistically significant and showed positive correlations (F/t = 6.64, 3.53, 4.15, 5.94, *p* < 0.05).

**Table 1:** Differential analysis of family process factors of online social anxiety (*n* = 2,373).

		***N***	**$\bar{x}\;\pm$s**	**F/T**	***P***
Only child	Yes	322	30.19 ± 10.95	−0.38	0.702
	No	2,051	30.93 ± 10.04		
Relationship with compatriots	Poor	29	42.67 ± 6.43	4.15	0.007
	Fair	317	35.13 ± 10.63		
	Good	1,705	29.72 ± 9.82		
Parent-child relationship	Poor	31	38 ± 17.35	3.53	0.031
	Fair	692	33.12 ± 8.85		
	Good	1,650	29.74 ± 10.38		
Family atmosphere	Poor	531	33.17 ± 10.54	5.94	0.003
	Fair	1,330	31.44 ± 9.13		
	Good	512	26.78 ± 11.27		
Parental supervision	Rarely	72	24.43 ± 10.72	2.58	0.078
	Occasionally	1,520	31.74 ± 9.37		
	Often	781	29.63 ± 11.31		
Inter-parental relations	Poor	161	35.13 ± 12.12	6.64	0.002
	Fair	640	33.83 ± 8.92		
	Good	1,572	29.17 ± 10.06		

### Multiple Linear Regression Analysis of Online Social Anxiety

The total score of the online social anxiety scale of university students was used as the dependent variable. The five family process factors of parental relationship, parental supervision, parent-child relationship, sibling relationship, and family atmosphere were used as independent variables for multiple linear regression analysis. First, the covariance test was conducted, the VIF values were all <10, and there was no covariance between the variables. Total anxiety score = 36.914−4.09 × good parental relationship−4.16 × good family atmosphere−3.42 × good sibling relationship, i.e., the total anxiety score was on average 4.09 points lower when comparing good and poor parental relationships. The average total anxiety score was 4.16 points lower when comparing good and poor family atmospheres. The average total anxiety score was 3.42 points lower when comparing good and poor sibling relationships (refer to Table 2).

**Table 2:** Multiple linear regression analysis of online social anxiety (*n* = 2,373).

**Dependent variable: Online social anxiety total score of Guangxi university students**					
	**Unstandardized coefficient**		**Standardization coefficient**	**t**	**Sig**
	**B**	**Standard error**	**Beta**		
(Constant)	36.914	1.446		25.521	0
Good parental relationship	−4.094	1.347	−0.191	−3.04	0.003
Good family atmosphere	−4.158	1.548	−0.169	−2.686	0.008
Good sibling relationship	−3.417	1.413	−0.151	−2.418	0.016

## Discussion and Suggestions

### Risk Factors and Protective Factors in Family Process Factors Affecting Online Social Anxiety of University Students

The results of the study showed that parental relationship, family atmosphere, and sibling relationship influenced Guangxi University students' online social anxiety, where parental friendship, sibling friendliness, and family harmony were protective factors for Guangxi University students. The opposite were risk factors. That is, the more parents punish and control when educating their children, the stronger the domestic violence environment, and the more family conflicts, the higher the incidence of university students' online social anxiety. On the contrary, good parental and sibling relationships and high intimacy in the family are protective factors to reduce the incidence of Guangxi University students' online social anxiety.

The risk factors among the family process factors that triggered or increased the online social anxiety of university students were poor parental relationships, poor sibling relationships in non-one-child families, and overall family climate disharmony. From the viewpoint of family systems theory, these risk factors occur in the coupled system (high conflict between spouses and strong violent environment) and sibling system (unfriendly sibling relationship), leading to children's overall perception of poor family climate playing a role in online social anxiety of university students' promoting effect. It is clear from this that the risky effects of negative family environments occurring in multiple family subsystems are significant, and it is evident that online social anxiety of university students is not the result of the action of a single system in the family but maybe the result of multiple risk systems acting together.

### Suggestions From the Perspective of Family Systems Theory

According to stress-coping theory, one of the main reasons that adolescents face a variety of risk factors during their development and only a minority of them end up with problems is that these risk factors do not act on the same adolescent at the same time. Adolescents' own psychological abilities (e.g., mental toughness, high self-efficacy, etc.) counteract the negative consequences of negative factors, a principle that is also valid for online social anxiety. If an adolescent grows up with only one risk factor, such as poor parental relationship or weak parental supervision, or sibling tension, the likelihood of online social anxiety in adolescents is low. However, when many risk factors, such as poor parental relationship, sibling disharmony, and family tension, act on adolescents at the same time, there is a high probability that online social anxiety will occur (28).

Developmental psychopathology suggests that the development of social adjustment problems in individuals is influenced by several factors that increase the risk of developing mental illness, while some factors protect individuals from physical and mental illness. Since the occurrence of online social anxiety is the result of a combination of risk factors, the more risk factors there are for online social anxiety interventions, the more protective factors there are to counteract the risk factors. The results of this empirical analysis of the factors influencing the online social anxiety of Guangxi University students showed that the total anxiety score of good parental relationships was on average 4.09 points lower than that of poor parental relationships. The average anxiety score of a good family atmosphere was 4.16 points lower than that of a poor family atmosphere. The average anxiety score of a good sibling relationship was 3.42 points lower than that of a poor sibling relationship, which were the protective factors of family process factors in reducing online social anxiety. A healthy developing adolescent has a positive support system, while a poorly developing adolescent has a negative support system behind him or her. In addition, there is a pattern of “good together and bad together” between the systems.

Although online social networking makes up for the deficiency of public real interpersonal communication to a certain extent. However, due to the virtuality, vulnerability, and uncertainty of the network, coupled with the proliferation of information, interpersonal overload, and privacy security of online social media, online social networking is mostly superficial and lacks depth, and personal emotional needs cannot be met, which promotes the emergence of online social anxiety and indulges it. As the most important place for emotional breeding and cultivation, the family should become a warm home to avoid and overcome the online social anxiety of university students. Based on the empirical analysis of this thesis, to intervene in the online social anxiety of university students, on the one hand, we need to intervene in the support system behind university students comprehensively. In terms of family process factors, we need to intervene in the coupled system, parent-child system, and sibling system in the family as a whole and improve the education and guidance mechanism of protective factors. On the other hand, when we intervene, we can also seize the key points and key links in the system. In the specific implementation method of correcting network anxiety through family, a generalized collaborative framework named collaborative shared healthcare plan (CSHCP) gives us a great idea to correct online social anxiety of university students (29). It is a CSHCP framework for daily life activity recognition and can be used for remote coordination and communication between family members to enhance emotion and avoid addiction to the network. This is more suitable for college students who leave their families to study. For college students with online social anxiety, their personal health records (PHRs) can be maintained using the Star File System (IPFS) (30), and they can access their records online and make targeted corrections at any time.

## Conclusion

Family is not only an extremely important environment in the process of individual psychological development and even the formation of psychological quality but also the main influencing factor of individual personality characteristics and mental health. Based on this, the thesis makes some tentative enquiries into the relationship between family process factors and online social anxiety. Although the statistical value of the sample survey cannot infer the overall trend quantitatively, the survey results of the sample can explain some overall characteristics: that parental relationship, family atmosphere, and sibling relationship influenced online social anxiety of university students. To intervene in the online social anxiety of university students, on the one hand, we need to intervene in the coupled system, parent-child system, and sibling system in the family as a whole and improve the education and guidance mechanism of protective factors. On the other hand, we can also concentrate on the key points and key links in the family process system. Because this study is affected by the randomness of the sampling survey and the limitation of the number of questionnaires, and the understanding of online social anxiety will be more or less subjective, the conclusion of the study is not suitable for popularization and needs further verification. Finally, it is hoped that through the relevant elaboration of the family factors of online social anxiety, the public can pay attention to family harmony.

## Data Availability Statement

The original contributions presented in the study are included in the article/supplementary material, further inquiries can be directed to the corresponding author/s.

## Ethics Statement

The studies involving human participants were reviewed and approved by Wei Wang, Wen Wu, Ling Zhang, Yun Lu, Qing Wang, Guangxi Police College. The patients/participants provided their written informed consent to participate in this study. Written informed consent was obtained from the individual(s) for the publication of any potentially identifiable images or data included in this article.

## Author Contributions

DB provided the overall idea of the article, the project design, and data analysis.

## Funding

This article titled Research on Social Integration of Migrant Children Relocated to Poverty Alleviation Areas in Guangxi was supported by the research project of Guangxi Philosophy and social sciences in the year 2021 (21FRK003). This article is part of the research results of the research project.

## Conflict of Interest

The author declares that the research was conducted in the absence of any commercial or financial relationships that could be construed as a potential conflict of interest.

## Publisher's Note

All claims expressed in this article are solely those of the authors and do not necessarily represent those of their affiliated organizations, or those of the publisher, the editors and the reviewers. Any product that may be evaluated in this article, or claim that may be made by its manufacturer, is not guaranteed or endorsed by the publisher.
